# Thermodynamic, Structural and Thermoelectric Properties of AgSbTe_2_ Thick Films Developed by Melt Spinning

**DOI:** 10.3390/nano8070474

**Published:** 2018-06-27

**Authors:** Baoli Du, Ming Liu, Jian Xu, Baofu Hu, Bingguo Liu, Taichao Su, Jian Wang

**Affiliations:** 1 School of Physics and Electronic Information Engineering, Henan Polytechnic University, Jiaozuo 454000, China; dbl@hpu.edu.cn (B.D.); 211711020001@home.hpu.edu.cn (M.L.); xujian@hpu.edu.cn (J.X.); wlhbf@hpu.edu.cn (B.H.); lbg@hpu.edu.cn (B.L.); 2 School of Material Science and Engineering, Henan Polytechnic University, Jiaozuo 454000, China; stc@hpu.edu.cn

**Keywords:** thermoelectric properties, AgSbTe_2_, thick films, rapid cooling, melt spinning, doping, microstructure

## Abstract

Cubic AgSbTe_2_ compound is a metastable phase within Ag_2_Te-Sb_2_Te_3_ pseudo-binary phase diagram and theoretically rapid cooling molten elements to room temperature may be an effective way to obtain it. In this work, thick films composed of 5–10 nm fine grains were developed by a melt spinning technique. The formation mechanism of the nanostructure and its influences on the thermoelectric properties have been studied and correlated. Differential scanning calorimetry (DSC) analysis shows that the as-prepared films exhibit distinct thermodynamic properties when prepared under different cooling rates and doping element. A small amount of Se doping is effectively capable of inhibiting the emergence of the Ag_2_Te impurity and optimizing the electrical transport properties. All films have positive large Seebeck coefficient, but rather small positive or negative Hall coefficient, indicating a multicarrier nature of transport consisting of both holes and electrons. A power factor of ~1.3 was achieved at 500 K for Se-doped film for its excellent electrical conductivities. This result confirms that a combination of Se doping and melting spinning technique is an effective way to obtain high phase-pure AgSbTe_2_ compound and reveal its intrinsic transport properties routinely masked by impurities in sintering or slow-cooling bulk samples.

## 1. Introduction

Thermoelectric materials are capable of converting waste heat from industry and vehicles into useful electricity [1]. Now most researchers are focused on the design and development of new thermoelectric material with high figure-of-merit *zT*, where *z* = *α*^2^*σ*/(*κ*) is determined by the interrelated materials parameters, Seebeck coefficient *α*, electrical conductivity *σ*, and thermal conductivity *κ*. Slack suggested that a good thermoelectric material would behave as a ‘phonon-glass, electron-crystal’, having high electrical conductivity and low thermal conductivity simultaneously [2]. Most of the strategies explored over the last decade were focused on minimizing the thermal conductivity while delicately avoiding detrimental effects on their electrical transport properties, for example, nanostructuring [3,4] and rattling guest atoms [5,6].

AgSbTe_2_ compound is not only the common end component of famous thermoelectric systems (AgSbTe_2_)_1−*x*_(GeTe)*_x_* [7] and (AgSbTe_2_)_1−*x*_(PbTe)*_x_* [3], but also has the highest figure-of-merit *zT* of all simple ternary compounds [4,8,9,10]. It has a large Seebeck coefficient and a low thermal conductivity, because of the complexity of ordering of Ag/Sb on the face-centered lattice and strong lattice vibrational anharmonicity [11,12,13]. In 2008, Wang et al. [10] reported a *zT* of 1.59 by mechanical alloying, and Du et al. [4] followed with a *zT* of 1.65 by a melt-spinning-spark-plasma-sintering synthesis route in 2011.

Though AgSbTe_2_ is a promising *p*-type TE material, its thermodynamic stability is still a controversial issue [14,15]. Based on the Ag_2_Te-Sb_2_Te_3_ pseudo-binary phase diagram, the cubic phase is a metastable phase only existing at high temperatures and prone to decompose to Ag_2_Te and Sb_2_Te_3_ below 633 K. Early reports revealed that samples obtained by melting and slow-cooling [14], zone melting [16], mechanical alloying [10], sonochemical method [17], hot pressing [18], as well as by high-pressure and high-temperature techniques [19], contain a small amount of precipitated Ag_2_Te, Ag_0.35_Sb_0.09_Te_0.56_, or other undesirable impurities. Meanwhile, transport properties studies showed it maintains a large positive Seebeck coefficient, while some samples have positive Hall coefficients and others have negative ones [11]. Clearly, these early materials might be inhomogeneous, and the intrinsic properties cubic AgSbTe_2_ compound were undetermined.

According to the phase diagram, the high temperature metastable cubic phase can only be achieved by non-equilibrium preparation route, for example, rapid cooling from molten phase to room temperature, and physical vapor deposition. Melt spinning is a rapid cooling technique (10^5^–10^6^ Ks^−1^) used to prepare materials that require extremely high cooling rates in order to form, such as metallic glasses [20]. It has been widely applied to thermoelectric systems to achieve nanostructured materials or materials with nano-crystalline properties [21,22]. However, this previous research were focused on the thermoelectric properties of the bulk materials sintering from thick films/ribbons obtained after melt spinning. Meanwhile, the reported transport properties of cubic AgSbTe_2_ compound varied from one research to another [4,8,9,10,11,12,14,15,17,18,19,23]. No efforts were put in the characterization of the melt-spun thick films itself. For AgSbTe_2_ compound, the thick films should possess a purer cubic phase than bulk sintered materials, which underwent at least one more heat treatment during the sintering with limited cooling rate. So there might be traces of impurities in the sintered samples, which is capable of masking the intrinsic transport of the cubic phase.

In this work, we tried to characterize the melt-spun thick film and reveal the intrinsic transport properties of cubic AgSbTe_2_. A combination of ultrahigh cooling rate and suitable dopant was used to try to develop phase-pure metastable AgSbTe_2_ and reveal its intrinsic transport. The formation mechanism of the nanostructure and its influences on the thermoelectric properties were studied and correlated. The effects of cooling rate and Se dopant on thermodynamic, structural, and transport properties were also investigated.

## 2. Materials and Methods

### 2.1. Film Prepartion

The starting materials for melt spinning were ingots obtained by quenching mixed molten high purity elements, Ag (99.995%, filament), Sb (99.9999%, shot), Se (99.95%, shot), and Te (99.999%, shot) in supersaturated brine. The ingot was then loaded into a quartz ampule with a 0.35 mm diameter nozzle at the bottom. The ampule was inserted into the melt spinning apparatus and re-heated, melted by an induction coil under the protection of an inert atmosphere. The melt was pressed out of the ampule under a pressure of 0.02 MPa and solidified in the form of thick films/ribbons on a chilled spinning copper roller. The rotation speed (linear speed of the outer edge) was adjusted to achieve different cooling rates. Due the fragility of the melt-spun films, the cooling rate and the completeness of the films were carefully balanced by choosing 5 and 10 ms^−1^ to develop materials with comparably low and high cooling rate, respectively.

### 2.2. Film Characterization

The constituent phases of the thick films were identified using powder X-ray diffraction (XRD, CuKa, X’Pert PRO-PANalytical, Almelo, Netherland) in the range of 5°–120°. The thermal properties were estimated by power compensation DSC (Q20, TA Instruments, New Castle, DE, USA) up to 673 K using a ramp rate of 20 Kmin^−1^ in a flow Ar atmosphere. The surfaces and cross section morphology were characterized using a field emission scanning electron microcopy (FESEM, S-4800, Hitachi, Tokyo, Japan) with energy dispersive X-ray spectroscopy (EDS). The inner microstructure was observed using an analytical high-resolution transmission electron microscopy (HRTEM, H-600 STEM/EDX PV9100, Hitachi, Tokyo, Japan). The specimen was thinned by ion bombardment using a Hitachi IM4000Plus ion milling system with cooling unit, and a typical plasma cleaning time of 10 min was applied. For AgSbTe_2_ film, which is very thermally sensitive, an intermittent milling procedure was used to achieve better heat dissipation with low ion energy at a milling angles of 6 degree. The electrical conductivity of AgSbTe_2_ films was measured using four-probe method on a homemade instrument in a vacuum up to 500 K. The fragile film was fixed by soft heat conducting silicone on an insulating ceramic substrate. Four electrodes were placed carefully by attaching 50 μm gold wires to the film using high-temperature silver paste. The contact resistance of each electrode is less than 1.0 Ω. The Seebeck coefficient was measured by a comparative method with Konstantan (Ni: 40%) as a reference sample in a vacuum. To minimize the influence of the thickness fluctuation and curvature of the film on electrical transport properties measurement, flat films with a typical dimension of 5 mm × 2 mm × 15 μm was used. The repeatability of the measurements was better than 5%, while the errors of resistivity and Seebeck coefficient are less than 8% and 5%, respectively. The films were rather robust and no apparent change were observed in the Seebeck coefficient and electrical conductivity when repeat measurements were done below 500 K. The Hall coefficient was measured by an Accent HL5500PC Hall measurement system (Accent, Bristol, UK) using the van der Pauw method with a magnetic field of 5340 G. For technical reasons, including the fragility and size of the films, no thermal conductivity data were collected. The figure-of-merit *zT* were roughly estimated, assuming that all film specimens had the same lattice thermal conductivity, with bulk sample prepared by a combination of melt spinning and spark plasma sintering.

## 3. Results and Discussion

### 3.1. Phase Structure, Thermodynamic Properties, and Microstructure

**Figure 1:**
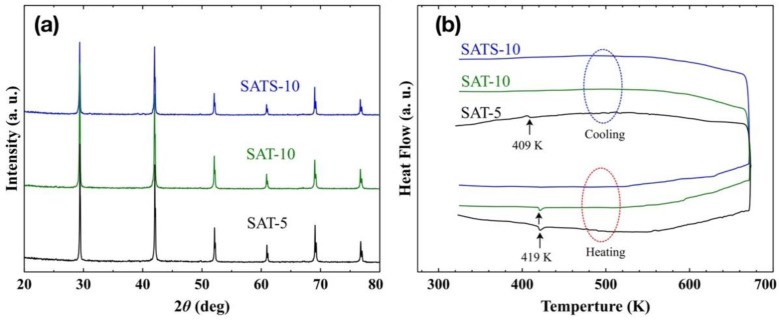
(**a**) Powder X-ray diffraction (XRD) patterns and (**b**) differential scanning calorimetry (DSC) heat flow curves of SAT-5, SAT-10 and SATS-10 films developed by melt spinning (sample mass, 10 mg).

Figure 1a shows the XRD patterns of both pristine AgSbTe_2_ thick films developed at linear speed of 5 and 10 ms^−1^, and Se-doped AgSbTe_1.98_Se_0.02_ film prepared at 10 ms^−1^. Selenium was chosen as a dopant because previous work displayed that 1% of Se is capable of inhibiting the formation of AgTe_2_ impurities and improving the thermoelectric properties in bulk samples prepared by spark plasma sintering [9,14]. To be clear, the three films were labeled as SAT-5, SAT-10, and SATS-10 in the following discussion, respectively. All the XRD peaks are well assigned to a cubic phase (fcc lattice, Fm$\bar{3}$m group, formula AgSbTe_2_) and no perceptible peaks with respect to known impurities were detected, such as AgTe_2_ and Ag_0.35_Sb_0.09_Te_0.56_. However, due to the subtlety of the Ag_2_Te-Sb_2_Te_3_ pseudo-binary phase diagram and limited test accuracy of XRD itself, the possibility of traces amount of impurity precipitation in the as-prepared films could not be ruled out completely.

**Figure 2:**
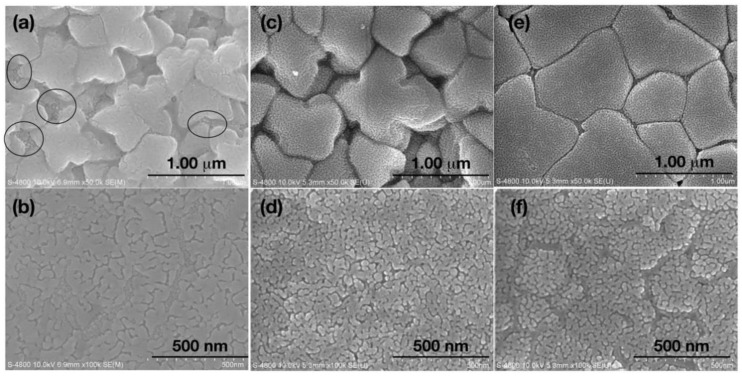
FESEM (field emission scanning electron microcopy) photographs of thick films developed by melt spinning. (**a**,**c**,**e**) are taken on the free side of films SAT-5, SAT-10 and SATS-10, respectively. (**b**,**d**,**f**) are taken on the wheel side of films SAT-5, SAT-10 and SATS-10, respectively. Small Ag_2_Te particles in image (**a**) were labeled by ovals. All images were collected on films without any processing or treatment after melt spinning.

Figure 2a,b displays the microstructure of both sides of the SAT-5 films, one is the wheel side where film had contact with copper wheel during solidifying from liquid, the other is the free side where grains were growing more freely during melt spinning. Dendritic patterns were observed on the wheel side of AST-5 film, suggesting that the cooling rate was not high enough to completely ‘freeze’ the grains nucleated on the surface of chilled copper wheel, resulting in the merge between the adjoining grains, and development of dendritic AgSbTe_2_. This result was unwelcome when we tried to achieve impurity-free cubic AgSbTe_2_ phase by rapid cooling. To make matters worse, small round particles marked by ovals in Figure 2a were observed between large grains on the free side. The appearance of these particles casts more doubt on the XRD results and led us to re-consider the phase structure of all the films.

In order to determine the phases of the obtained films accurately, the DSC analysis was performed and the heat flow curves are shown in Figure 1b. For SAT-5 film, endothermic and exothermic peaks corresponding to the α–β phase transition of Ag_2_Te were detected at 419 and 409 K during both heating and cooling processes, respectively [24,25]. This suggests that the small particles around large grains on free side were α-Ag_2_Te precipitations, and the impurity cannot be integrated into the AgSbTe_2_ matrix during heat treatment up to 673 K. For SAT-10 film achieved at higher cooling rate, the DSC curve only revealed an endothermic peak at 419 K in the heating process, while the exothermic peak was disappeared completely in the cooling process. Surely, the SAT-10 film consists of traces of α-Ag_2_Te impurity. However, it dissolved into the matrix gradually in the heating process irreversibly, which means that the SAT-10 film has rather homogeneous element distribution, though weak phase separation occurred during the formation process. This analysis was consistent with the EDS results obtained on both the wheel side and free side. The actual composition of SAT-10 film varied within 2% of the nominal one, and fell well within the expected range. Thus, a linear speed of 10 ms^−1^ was used to prepare Se-doped film. The thickness of the melt-spun film decreased with the increase of the linear speed of the copper wheel, and further increase of the linear speed damaged the completeness of the film, and generated large portions of dust-like materials.

No peaks were observed during DSC measurement, and the result suggests that the SATS-10 film is a phase-pure cubic AgSbTe_2_ compound free from impurity Ag_2_Te. That implies that 1% Se is capable of preventing the precipitation of the α-Ag_2_Te, just as it does in the melt-quench-spark-plasma-sintering bulk samples [9]. Early research reported that partial substitution of Te by Se leads to the stabilization of the cubic structure of the AgSbTe_2_-AgSbSe_2_ pseudo-binary system [14]. However, the DSC result revealed multiple peaks corresponding to the existence of both Ag_2_Te, Ag_0.35_Sb_0.09_Te_0.56_, and possible other precipitations in the samples prepared by melt-slow-cooling route. So both a high cooling rate and suitable dopant were crucial to develop the high purity cubic AgSbTe_2_ phase.

As shown in Figure 2c,d, 400–600 nm grains with clear boundaries were observed on the free side of the SAT-10 film. On the wheel side, 20–100 nm nano-grains are packed tightly with no signs of any dendritic pattern. Interestingly, 20–40 nm fine surface texture and pores/voids in between grains were observed on the wheel side grain surface. These features are similar with SAT-5 film. The solo difference is that the SAT-10 film is free of perceptible AgTe_2_ particles at the grain boundaries due to the higher cooling rate. Figure 2e,f shows FESEM photographs of the free side (e) and wheel side (f) of the SATS-10 film. Unlike the pristine films SAT-5 and SAT-10, the wheel side of Se-doped film looks rather flat, free of pores and voids in between grains. All grains with fine surface texture were packed tightly as the morphology of the wheel side of two pristine films, if the grain boundaries were neglected. This suggests that Se doping has significant influences on the grain growth and restrains the free growth of grains on the free side toward the direction perpendicular to the surface of the film. So the grains have to expand and grow parallel to the surface, which leads to the flat features of the free side. The influence is also reflected in the wheel side image. Unlike the homogeneous distribution of grains in SAT-10 film, gully-like structures were formed on the wheel side due to the strain accompanying the refrain of free growth of the grains on the opposite side.

**Figure 3:**
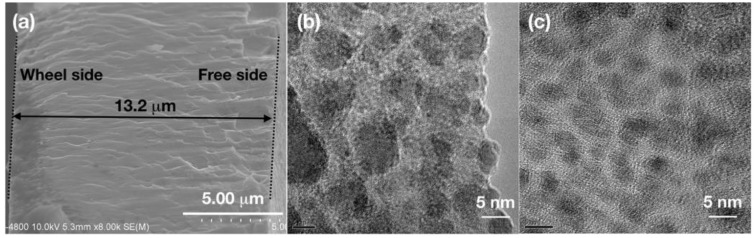
HR-TEM (high-resolution transmission electron microscopy) micrographs of thick films (**b**), SAT-10; (**c**), SATS-10 developed by melt spinning. A cross section SEM image of film SAT-10 (**a**), was placed to help readers to have an overall understanding of the thick films. Both TEM specimen were thinned from melt-spun films using ion bombardment without any other processing or treatment.

To have an overall understanding of the melt-spun films. The cross section of SAT-10 film is displayed in Figure 3a. The film had clear boundary on both wheel side and free side, and a thickness of about 13.2 μm was measured for SAT-10 film. TEM images of SAT-10 and SATS-10 films are shown in Figure 3b,c, which display the structural details of the interior of the films by exfoliate the surface layer using ion bombardment. Both SAT-10 and SATS-10 films displayed fine nanostructure. In addition to the 20–100 nano-grains on the wheel side and 20–40 nm grain surface texture on the free side disclosed by SEM, grains as small as 5–10 nm were observed in the TEM image of film SAT-10. This suggests that the cooling rate was so huge that grains ranging from 5–100 nm were frozen during melt spinning. The SATS-10 film showed an even finer and more homogeneous nanostructure. The observed fine grains as small as 5 nm among a likely amorphous background indicated that the films might have inherited part of the structural character of the molten phase due to the ultrahigh cooling rate. This result is corresponding to the analysis on SEM image and confirms that Se have substantial influence on the growth of AgSbTe_2_ thick films. We conjectured that bulk samples synthesized by combination use of melt spinning and spark plasma sintering, benefits a lot from the fine and hieratical microstructure of AgSbTe_2_ thick films. So the low lattice thermal conductivity is not only related to the nanopores on the grain surface in the bulk sample, but also owing to the fine and hieratical microstructure in the melt-spun thick films/ribbons [4].

### 3.2. Transport Properties

**Figure 4:**
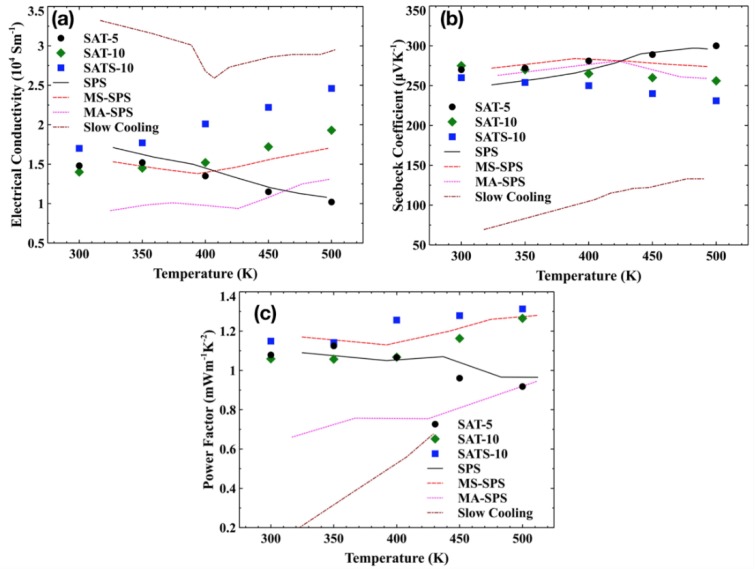
Temperature dependence of (**a**) the electrical conductivity, (**b**) the Seebeck coefficient, (**c**) the power factor of SAT-5 (black circle), SAT-10 (green diamond) and SATS-10 (blue square) thick films developed by melt spinning. For comparison, reference samples prepared by spark-plasma-sintering (SPS, black solid line) [26], melt-spinning-spark-plasma-sintering (MS-SPS, red dash line) [4], mechanical-alloying-spark-plasma-sintering (MA-SPS, magenta dotted line) [10] and melt-slow-cooling (Slow cooling, dark red dash-dot line) [14] were also included.

The temperature dependence of the conductivity *σ* and the Seebeck coefficient *α* for melt-spun films and reference samples are shown in Figure 4a,b. All films had similar electrical conductivities at room temperature. However, the evolution trend of electrical conductivity upon temperature varied from film to film. With the increasing temperature, electrical conductivity of SAT-5 film decreased steadily, while these of SAT-10 and SATS-10 had the opposite trend. Moreover, for the SAT-10 film, the electrical conductivity showed an apparently weaker dependence of temperature below 400 K and a stronger one above 400 K. Combined with the phase structure elaborated in Section 3.1 and the abnormal behaviors of samples reported in the literature [4,9,10,14,16,19,25,26], we deduced that the contrasting conductivity features are closely associated with Ag_2_Te precipitates in the films. A small amount of Ag_2_Te had strong influence on the transport properties of film SAT-5 and covered the intrinsic character of AgSbTe_2_ completely. Meanwhile, traces of Ag_2_Te impurities changed the temperature dependence of electrical conductivity of film SAT-10 below 400 K. However, the influences diminished gradually above 400 K because of the merging of Ag_2_Te impurities with the matrix in SAT-10. This result is in accord with the DSC analysis on the thermodynamic properties film SAT-10. So, only the film SATS-10 film exhibits the intrinsic properties of AgSbTe_2_ compound in the whole characterization temperature range. No reference samples are immune to the influences from Ag_2_Te precipitate. The above discussion confirmed that the Ag_2_Te precipitate in AgSbTe_2_ has strong influences on the transport properties, and traces of it is enough to cover the intrinsic properties of AgSbTe_2_ compound.

All films exhibited large positive Seebeck coefficients ranging from 230 to 300 μVK^−1^, comparable to the values achieved in SPS, MS-SPS and MA-SPS bulk samples. As expected, samples with low electrical conductivity had higher Seebeck coefficients and vice versa. Moreover, a similar bifurcation behavior was observed in the Seebeck coefficient, similar with what we have seen in the case of electrical conductivity.

To gain more insight into the electrical transport behavior, Hall effect measurements were carried out at room temperature. The results are presented in Table 1. All films show very small Hall coefficient, and the value changes from positive one for Se-doped SATS-10 film to negative one for pristine SATS-5, and SAT-5 films. According to the phase structure and electron band structure, there are two factors contributing to the abnormality of Hall coefficient. One is the *n*-type impurity Ag_2_Te, the other is the distinct transport nature of the AgSbTe_2_ compound itself, in which both electrons and holes take part in the charge transport [11].

When regarding the SAT-5 and SAT-10 films as composites between cubic AgSbTe_2_ and α-Ag_2_Te, the small amount of *n*-type impurity is far from enough to shift the Hall coefficient from positive to negative. So, the Seebeck coefficient and Hall coefficient can be described by formulae (1) and (2) as in a typical two-carrier system, where the Seebeck coefficient is the sum of first-order terms in the carrier mobility, whereas the Hall coefficient is the sum of quadratic terms in the carrier mobility [11].

(1) $$\alpha =\frac{n\alpha_{e}\mu_{e}+p\alpha_{h}\mu_{h}}{n\mu_{e}+p\mu_{h}}$$

(2) $$R_{H}=\frac{1}{q}\frac{p\mu_{h}^{2}-n\mu_{e}^{2}}{{\left(n\mu_{e}+p\mu_{h}\right)}^{2}}=\frac{1}{q}\frac{p-nb^{2}}{{\left(p+nb\right)}^{2}}$$

Here, *p* and *n* are the electron and hole concentrations; *μ_e_* and *μ_h_* are the electron and hole mobilities; *α_e_* and *α_h_* are the partial electron and hole Seebeck coefficients, and *b* is the ratio between hole and electron mobilities (*b* = *μ_e_*/*μ_h_*). So, the Hall coefficient can have an opposite sign with respect to the Seebeck coefficient when the mobility of electrons (minority-carrier) is much higher than the mobility of holes (majority carrier). Negative Hall coefficients have been observed in Se-doped AgSbTe_2_ samples prepared by spark plasma sintering and other methods [9,11]. Meanwhile, Jovovic and Heremans found that the mobility of electrons and hole are 2200 and 11 cmV^−1^s^−1^, respectively, with the *b* value as high as 200 [11]. So, both pristine films have small negative Hall coefficients. However, the 1% Se-doping shifted the Hall coefficient of SATS-10 film to a small positive one. Based on the formula (2), an increase of hole concentration *p* or a decrease of mobility ratio *b* are capable of shifting the Hall coefficient from negative to positive. In AgSbTe_2_, Selenium is an acceptor-type dopant and can increase the hole concentration *p* [9]. Also, point defects accompanying the incorporation of Selenium in the Tellurium sublattice enhance the scattering of the charge carrier, especially the high-mobility minority-carrier electron, and decrease the difference between the two mobilities (decrease *b*). Though it is impossible to quantify the contributions of each factor. We are confident that the Se has both substantial impact on the nature of transport in AgSbTe_2_ films.

Figure 4c presents the temperature dependence of power factor *α*^2^*σ* for films and reference bulk samples. Both SAT-10 and SATS-10 film have very high power factors compared with bulk samples, owing to their excellent electrical conductivities. A power factor of ~1.3 was achieved at 500 K for film SATS-10. Due to the fragility and size of the films, no thermal conductivity data were collected. In semiconductors, the total thermal conductivity *κ* can be written as *κ* = *κ*_latt_ + *κ*_carr_, where *κ*_latt_ and *κ*_carr_ are the lattice and carrier contributions, respectively. The carrier component can be calculated using the Wiedemann-Franz law as *κ*_carr_ = *LTσ,* where the Lorenz number *L* = 0.7 *L*_0_, *L*_0_ equals 2.45 × 10^−8^ V^2^/K^2^ for the fully degenerate semiconductor. Assuming the *κ*_carr_ is constant for all films and bulk samples prepared by MS-SPS, the *κ* value for film samples can be given by a formula, *κ* = *κ*_latt_ (bulk sample) + *κ*_carr_. Here, we roughly estimated the dimensionless figure-of-merit *zT*s based on the measured values of Seebeck coefficients, electrical conductivity, and calculated data of thermal conductivity. A *zT* of 1.40 was obtained by film SAT-10 at 500 K, which is higher than any other reference samples at the same temperature. Though the maximum *zT* of 1.35 of film SATS-10, estimated using same lattice thermal conductivity, is lower than that of SAT-10 at 500 K, it may have a higher *zT* value when taking into account of the effect of point defect scattering on phonon transport accompanying with the substitution of Te by Se, which has been well demonstrated in bulk AgSbSe*_x_*Te_2−*x*_ samples prepared by SPS [9]. This suggest that reviving the intrinsic properties of the cubic AgSbTe_2_ compound and refraining the precipitation of Ag_2_Te impurity by Se doping and repaid cooling are potential ways to optimize the thermoelectric properties.

## 4. Conclusions

Rather than focusing on bulk sintered samples, we investigated the microstructure and transport properties of AgSbTe_2_ thick films developed by melt spinning, and discussed the effects of Se doping and cooling rate on their structural, thermodynamic, and transport properties. All films possess fine nanostructures, with 400–600 nm grains composed of 20–40 nm fine surface texture on the free side, and 20–100 nm ones on the wheel side. TEM observed fine grains as small as 5 nm among a likely amorphous background, indicating that the films might have inherited part of the structural characters of the molten phase, and formed a 5–100 nm hieratical microstructure due to the ultrahigh cooling rate. Pristine films developed under different cooling rate, and Se-doped film exhibit distinct thermodynamic properties during DSC measurement. Tiny peaks corresponding to the α–β phase transition of Ag_2_Te compound manifested themselves during both DSC heating and cooling process for the SAT-5 film, while they only appeared in heating process in SAT-10 film obtained under higher cooling. Transport properties measurements suggest that both holes and electrons contribute to the electrical properties, and pristine films and Se-doped film have opposite sign of the Hall coefficient, while all films have large positive Seebeck coefficients. Films with Se doping are completely free of the impurity Ag_2_Te, and shows the intrinsic transport properties of the cubic AgSbTe_2_ compound. This result suggests that it is possible to obtain nanostructured phase-pure metastable compounds, and reveal its intrinsic transport properties by combined use of ultrahigh cooling rates and suitable dopants.

## Figures and Tables

**Table 1:** Hall coefficients of thick films developed by melt spinning.

Films	SAT-5	SAT-10	SATS-10
Hall coefficient 10^−8^ m^3^ C^−1^	−5.7	−3.9	2.7
